# Transcriptional and functional characterization of genetic elements involved in galacto-oligosaccharide utilization by *Bifidobacterium breve* UCC2003

**DOI:** 10.1111/1751-7915.12011

**Published:** 2012-12-02

**Authors:** Mary O'Connell Motherway, Michael Kinsella, Gerald F Fitzgerald, Douwe Sinderen

**Affiliations:** 1 Alimentary Pharmabiotic Centre, National University of IrelandWestern Road, Cork, Ireland; 2 Departments of Microbiology, National University of IrelandWestern Road, Cork, Ireland; 3 Food and Nutritional Sciences, National University of IrelandWestern Road, Cork, Ireland

## Abstract

Several prebiotics, such as inulin, fructo-oligosaccharides and galacto-oligosaccharides, are widely used commercially in foods and there is convincing evidence, in particular for galacto-oligosaccharides, that prebiotics can modulate the microbiota and promote bifidobacterial growth in the intestinal tract of infants and adults. In this study we describe the identification and functional characterization of the genetic loci responsible for the transport and metabolism of purified galacto-oligosaccharides (PGOS) by *Bifidobacterium breve* UCC2003. We further demonstrate that an extracellular endogalactanase specified by several *B. breve* strains, including *B. breve* UCC2003, is essential for partial degradation of PGOS components with a high degree of polymerization. These partially hydrolysed PGOS components are presumed to be transported into the bifidobacterial cell via various ABC transport systems and sugar permeases where they are further degraded to galactose and glucose monomers that feed into the bifid shunt. This work significantly advances our molecular understanding of bifidobacterial PGOS metabolism and its associated genetic machinery to utilize this prebiotic.

## Introduction

Neonates possess an immature intestinal immune system, a gastrointestinal tract devoid of a microbiota and a stomach with a relatively high pH, which is not effective in eliminating pathogens, thereby making newborns highly susceptible to infection. Colonization of the human gut commences at birth, when the mother's vaginal and faecal microbiota are considered to be the most important inocula (Vaishampayan *et al*., 2010). It has furthermore been suggested that mother's milk serves as an inoculum for breastfeeding infants as bacteria from the maternal gut are believed to reach the mammary gland through an endogenous route, the so-called ‘entero-mammary pathway’ that involves maternal dendritic cells and macrophages (Martín *et al*., 2003; Perez *et al*., 2007). Evidence is accumulating on the importance of the human gut microbiota in educating the immune system at this very early stage of extrauterine life (Martín *et al*., 2010). There is a broad consensus that breastfeeding is the best option for optimal development of the infant's intestinal microbiota, so as to promote a healthy, balanced immune system (reviewed by Marques *et al*., 2010). Human milk is a functional food; it contains semi-essential nutrients, free amino acids, enzymes, hormones, growth factors, polyamines, nucleotides and oligosaccharides (Walker, 2007b). Research has indicated that human milk has a protective effect against diarrhoeal diseases, respiratory tract infections, bacteremia, meningitis and necrotizing enterocolitis, all presumed to be mediated through its bioactive components (Martín *et al*., 2010). Human milk oligosaccharides (HMOs) are the main prebiotic factors in human milk and, while they do not directly nourish infants, HMOs are thought to enrich for specific gut commensals capable of utilizing these diverse substrates, in particular bifidobacteria that are abundant in and characteristic of the breastfed baby's intestinal microbiota (Sela and Mills, 2010; Sela, 2011).

Bifidobacteria are anaerobic, saccharolytic, high GC-content, Gram-positive bacteria that are non-motile and do not sporulate or produce gas through their fermentative metabolism (reviewed by Turroni *et al*., 2011). Bifidobacteria are frequently reported as being numerically dominant in the faecal microbiota of breastfed infants while their numbers decline on weaning and thereafter resemble those of the adult microbiota. The many beneficial effects associated with a breastfed infant microbiota, being high in bifidobacterial numbers, has resulted in major efforts being directed at manipulating infant food formulations so as to achieve in bottle-fed infants an intestinal microbiota composition resembling that found in breastfed babies. HMOs are very diverse in composition and structure (Niñonuevo *et al*., 2006; Lo Cascio *et al*., 2009; Niñonuevo and Lebrilla, 2009; Nwosu *et al*., 2012) and as yet these are not available for incorporation into infant food formulations. Consequently research has focused on the development of alternative prebiotic, non-human milk-derived oligosaccharides, such as galacto-oligosaccharides (GOS).

GOS are non-digestible oligosaccharides that are produced by enzymatic transglycosylation of lactose using β-galactosidases, thereby generating a mixture of oligomers of varying chain length ranging from DP2 to DP15 (Barboza *et al*., 2009). The raw material for the production of GOS is whey-derived lactose which is a by-product of the dairy industry. The composition of GOS is dependent on the source of the enzyme, substrate concentration and pH, furthermore, since GOS production involves a transglycosylation process which competes with the hydrolytic activity of the enzymes, GOS always contain a significant amount of lactose as well as its constituent monosaccharides galactose and glucose (Coulier *et al*., 2009).

Since 2002, several infant formulations containing specific mixtures of short-chain GOS (scGOS) and long-chain fructo-oligosaccharides (lcFOS) have been on the market. These formulations have been designed to elicit a prebiotic/bifidogenic effect comparable to that of human milk, as demonstrated in numerous clinical trials (reviewed by MacFarlane and Macfarlane, 2007; Sangwan *et al*., 2011). Furthermore, there is an increasing body of evidence that prebiotic supplementation of food formulations with GOS provides protection against pathogen infection (Eversbach *et al*., 2010; Searle *et al*., 2010; Quintero *et al*., 2011).

To date several β-galactosidases of bifidobacterial origin have been described in the literature and much research has focused on the ability of these enzymes to hydrolyse and synthesize GOS (Van Laere *et al*., 2000; Barboza *et al*., 2009; Goulas *et al*., 2009; Osman *et al*., 2010; 2012). In the present study we evaluated the transcriptional response of the human (infant) gut commensal *Bifidobacterium breve* UCC2003 during growth on a purified Vivinal® GOS (PGOS) formulation that contains very low levels of monosaccharides and lactose. Our results indicate that PGOS metabolism by *B. breve* UCC2003 is the result of the concerted action of several β-galactosidases and their associated transport systems that internalize GOS components. We furthermore demonstrate that an extracellular endogalactanase specified by several *B. breve* strains, including *B. breve* UCC2003, is essential for metabolism of GOS components with a long retention time and high degree of polymerization. The resulting endogalactanase-mediated breakdown products are transported into the bifidobacterial cell via various ABC transport systems and permeases where they are further metabolized to galactose and glucose, the latter feeding directly into the so-called bifid shunt, while the former likely being channelled through the Leloir pathway and converted to glucose 6-phosphate before entering the bifid shunt.

## Results

### PGOS utilization by *B. breve* strains

**Fig. 1:**
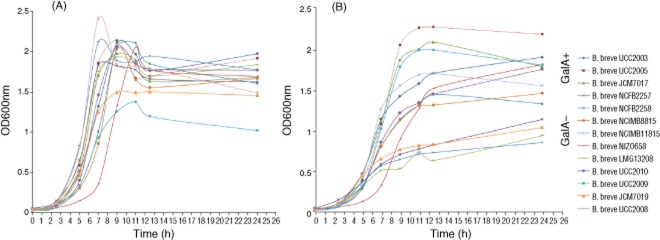
Growth profiles of *B. breve* strains on lactose (A) and PGOS (B). Data presented are averages of duplicate growth experiments.

**Fig. 2:**
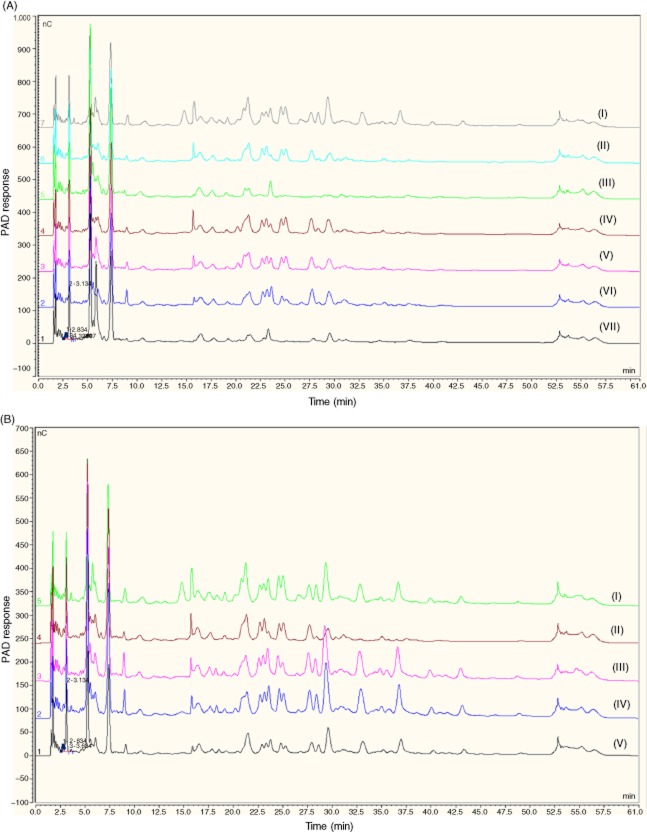
HPAEC-PAD analysis of post-fermentation cell-free supernatants of *B. breve* strains. Modified Rogosa supplemented with 0.5% GOS (AI and BI); post-fermentation supernatants of *B. breve* UCC2003 (AII and BII), endogalactanase positive strains: *B. breve* UCC2005 (AIII); NCFB2258 (AIV); NCIMB8815 (AV); NIZO658 (AVI) and UCC2008 (AVII) and endogalactanase negative strains: *B. breve* NCFB2257 (BIII); NCIMB11815 (BIV) and LMG13208 (BV).

We previously established that not all *B. breve* strains possess endogalactanase activity and by comparative genome hybridization were able to establish that the presence of endogalactanase activity is strictly correlated with the presence of the *gal*A gene in the *B. breve* strains examined (O'Connell Motherway *et al*., 2011b). In order to investigate the importance of endogalactanase activity for PGOS metabolism, growth of 13 *B. breve* strains, namely UCC2003, UCC2005, JCM7017, JCM7019, NCFB2257, NCFB2258, NCIMB8815, NCIMB11815, LMG13208, NIZO658, UCC2008, UCC2009 and UCC2010, was profiled in Modified Rogosa with 0.5% lactose or PGOS as the sole carbohydrate source. All strains grew well on lactose reaching an OD 600nm ≥ 1.0 within 8 h of growth, while growth on PGOS separated the *B. breve* strains into two groups: the first group all grew well on PGOS reaching an OD 600nm ≥ 1.0 within 9 h of growth while for the second group a final OD 600nm of ≤ 1.0 was achieved in the 24 h of the growth profile (Fig. 1). These two distinct bifidobacterial growth profiles correlated with the endogalactanase-positive (GalA^+^) and endogalactanase-negative (GalA^−^) strains respectively. Post-fermentation, cell-free supernatants were analysed by HPAEC-PAD and demonstrated that the GalA^+^ *B. breve* strains utilized almost all of the GOS components (Fig. 2A), while the GalA^−^ strains exhibited more variability in the GOS fractions that were metabolized. The GalA^−^ strains did not utilize PGOS components with a long retention time (between 30 and 48 min), which represent galacto-oligosaccharides with a high degree of polymerization (DP) (Coulier *et al*., 2009) (Fig. 2B), indicating that GalA is responsible for the extracellular degradation of these high DP GOS components.

### Genome response of *B. breve* UCC2003 to growth on PGOS

**Fig. 3:**
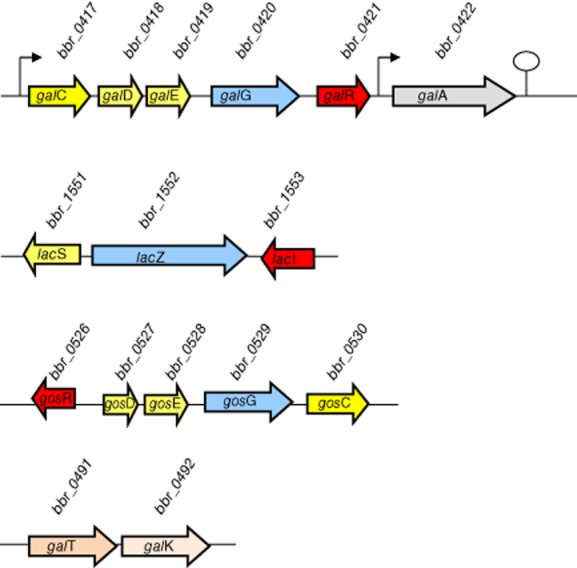
Schematic representation of *B. breve* UCC2003 gene clusters upregulated during growth of PGOS as sole carbohydrate source. The lengths of the arrows are proportional to the length of the predicted ORF and the gene locus name, which is indicative of its putative function, is indicated within the arrow. The bent arrows indicate the *gal*C and *gal*A promoters; the lollipop sign designates putative *rho*-independent terminator region. β-Galactosidase-encoding genes are indicated by blue shading, while genes encoding proteins with transport functions are shaded in yellow. Putative or proven genes encoding LacI-type transcriptional regulators are indicated by red shading.

In order to investigate which genes may be involved in PGOS metabolism in *B. breve* UCC2003, global gene expression was determined by microarray analysis during growth of this bifidobacterial strain on PGOS and this was compared with its expression pattern when grown on ribose as sole carbohydrate source. Total RNA was isolated from mid-log phase cultures of *B. breve* UCC2003 grown on PGOS or ribose as the sole carbohydrate source. Analysis of the DNA microarray data revealed that the expression of four gene clusters was significantly upregulated when UCC2003 was grown on PGOS (fold change > 3.0, *P* < 0.001; Table 1 and Fig. 3). The first GOS-inducible cluster of six genes, *gal*C, *gal*D, *gal*E *gal*G, *gal*R and *galA*, constitute the previously identified galactan metabolism cluster (O'Connell Motherway *et al*., 2011b), while the second cluster, Bbr_1551 to Bbr_1553, consists of three genes specifying a predicted galactoside symporter and a GH2 family β-galactosidase in divergent orientations, designated here as *lac*S and *lac*Z respectively, and an associated gene encoding a putative LacI-type transcriptional regulator. The third GOS-inducible cluster comprises five genes, Bbr_0526 to Bbr_530, that are predicted to specify a LacI-type transcriptional regulator, two ABC-type permeases, a GH42 family β-galactosidase and a solute-binding protein, which are designated *gos*R, *gos*D, *gos*E *gos*G and *gos*C respectively. The fourth set of upregulated genes are the presumed *gal*T and *gal*K genes that encode Galactose-1-phosphate uridyltransferase and galactokinase respectively (Kitaoka *et al*., 2005; Fushinobu, 2010), and represent key enzymes of the Leloir pathway that converts galactose to d-glucose 6-phosphate that can feed into the bifid shunt (Fushinobu, 2010; Pokusaeva *et al*., 2011a) (Fig. 3).

**Table 1:** Effect of PGOS on the transcriptome of *B. breve* UCC2003

Locus tag_gene	Putative function	PGOS^a^
Bbr_0417_*gal*C	Solute-binding protein	285.61
Bbr_0418_*gal*D	Sugar permease protein	9.08
Bbr_0419_*gal*E	Sugar permease protein	10.17
Bbr_0420_*gal*G	β-Galactosidase GH 42 family	99.99
Bbr_0421_*gal*R	Transcriptional regulator, LacI family	6.43
Bbr_0422_*gal*A	Endogalactanase	45.25
Bbr_1551_*lac*S	Galactoside symporter	174.68
Bbr_1552_*lac*Z	β-Galactosidase GH 2 family	128.14
Bbr_1553_*lac*I	Transcriptional regulator, LacI family	2.9
Bbr_0526_*gos*R	Transcriptional regulator, LacI family	5.62
Bbr_0527_*gos*D	Sugar permease protein	8.75
Bbr_0528_*gos*E	Sugar permease protein	9.99
Bbr_0529_*gos*G	β-Galactosidase GH 42 family	6.33
Bbr_0530_*gos*C	Solute-binding protein	5.83
Bbr_0491_*gal*T	Galactose-1-phosphate uridyltransferase	3.51
Bbr_0492_*gal*K	Galactokinase	3.22

a Expression ratios presented have a Bayesian *P*-value < 0.001 according to the Cyber-*T*-test (Long *et al*., 2001).

### Phenotypes of mutants carrying disruptions in the *lac*S, *lac*Z, *gos*D or *gos*G genes

**Fig. 4:**
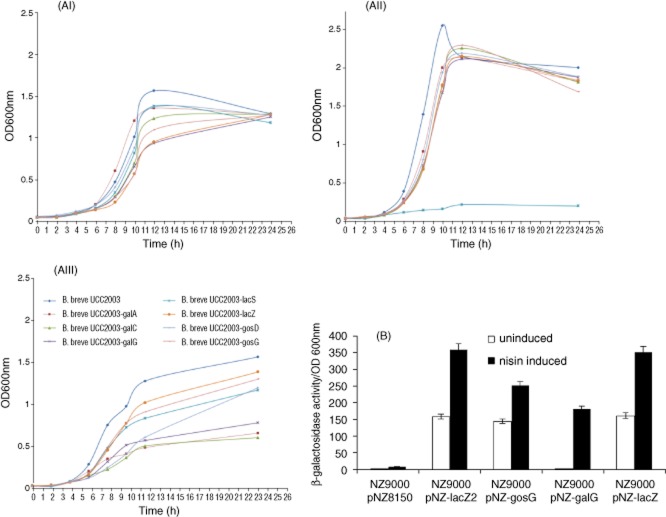
Growth profiles of *B. breve* UCC2003 and insertion mutant strains on ribose (AI), lactose (AII), and PGOS (AIII). Data presented are averages of duplicate growth experiments. B. β-Galactosidase activity assays of uninduced and nisin induced *L. lactis* cultures, NZ9000-pNZ8150, NZ9000-pNZ-lacZ2, NZ9000-pNZ-gosG, NZ9000-pNZ-galG and NZ9000-pNZ-lacZ.

In order to determine how disruption of particular genes from the *gal*CDEGR, *lac*SZI and *gos*RDEGC gene clusters would impact on the ability of *B. breve* UCC2003 to metabolize PGOS, the previously constructed mutant strains *B. breve* UCC2003-galA, UCC2003-galC and UCC2003-galG were employed (O'Connell Motherway *et al*., 2011b). Additional insertion mutants in the *B. breve lac*S, *lac*Z, *gos*D and *gos*G genes were generated, resulting in strains *B. breve* UCC2003-lacS, UCC2003-lacZ, UCC2003-gosD and UCC2003-gosG respectively (Table 2). To determine the components of PGOS that were metabolized by each of these insertion mutants as compared with *B. breve* UCC2003, strains UCC2003 (wild type), and these seven insertion mutant strains were analysed for their ability to grow in mMRS supplemented with PGOS, lactose, lactulose or ribose (positive control) as the sole carbon source and the cell-free, post-fermentation supernatants of PGOS fermentation were analysed for remaining PGOS fractions by HPAEC-PAD. As expected, and in contrast to the wild-type, the *B. breve* UCC2003-lacS insertion mutant was shown to be incapable of growth on lactose (Fig. 4AII) or lactulose (results not shown) as the sole carbon source thereby identifying LacS as the main lactose/lactulose transporter in this strain. Intriguingly, *B. breve* UCC2003-lacZ is capable of growth on lactose (Fig. 4AII) and lactulose (results not shown) as the sole carbohydrate source provided. This growth could be attributed to the presence of one or more additional β-galactosidases that can hydrolyse lactose in the absence of functional LacZ. Candidates for such alternative β-galactosidase-encoding genes are *lac*Z2, which is predicted to encode a GH2 family β,1-4-galactosidase, *gos*G or *gal*G (O'Connell Motherway *et al*., 2011b). To test this possibility these four prospective β-galactosidases, LacZ2, GosG, GalG and LacZ, were expressed in *Lactococcus lactis* NZ9000 using the nisin-inducible expression system (de Ruyter *et al*., 1996) after which β-galactosidase activity was measured in cell lysates of the various strains. Following nisin induction, cells of *L. lactis* NZ9000 expressing each of the four predicted bifidobacterial β-galactosidases exhibited significantly increased β-galactosidase activity thereby demonstrating that LacZ2, GosG, GalG and LacZ, can indeed hydrolyse lactose (Fig. 4B). The observed growth of the UCC2003-lacZ mutant strain in mMRS supplemented with lactose as the sole carbohydrate source is therefore likely due to the activity of one or more of the β-galactosidase enzymes specified by *lac*Z2, *gos*G or *gal*G.

**Table 2:** Bacterial Strains and Plasmids used in this study

Strain or plasmid	Relevant features	Reference or source
Strains		
*Escherichia coli* strains		
*E. coli* EC101	Cloning host, repA^+^ km^r^	Law *et al*. (1995)
*E. coli* EC101-pNZ-M.BbrII + M.BbrIII	EC101 harbouring pNZ8048 derivative containing *bbrIIM* and *bbrIIIM*	O'Connell Motherway *et al*. (2009)
*Bifidobacterium* sp. strains		
*B. breve* UCC2003	Isolate from nursling stool	Maze *et al*. (2007)
*B. breve* UCC2003-pBC1.2	UCC2003 harbouring pBC1.2	This study
*B. breve* UCC2003-galA	pORI19-tet-galA-967 insertion mutant of UCC2003	O'Connell Motherway *et al*. (2011b)
*B. breve* UCC2003-galA-pBC1.2-galA	pORI19-tet-galA-967 insertion mutant of UCC2003 harbouring complementationconstruct pBC1.2-galA	This study
*B. breve* UCC2003-galG	pORI19-tet-galG-410 insertion mutant of UCC2003	O'Connell Motherway *et al*. (2011b)
*B. breve* UCC2003-galC	pORI19-tet-galC-701 insertion mutant of UCC2003	O'Connell Motherway *et al*. (2011b)
*B. breve* UCC2003-gosD	pORI19-tet-bbr_0527 insertion mutant of UCC2003	This study
*B. breve* UCC2003-gosG	pORI19-tet-bbr_0529 insertion mutant of UCC2003	This study
*B. breve* UCC2003-lacS	pORI19-tet-bbr_1551 insertion mutant of UCC2003	This study
*B. breve* UCC2003-lacZ	pORI19-tet-bbr_1552 insertion mutant of UCC2003	This study
*B. breve* UCC2005	Isolate from human faeces	UCC
*B. breve* JCM7017	Isolate from human faeces	JCM
*B. breve* JCM7 019	Isolate from infant faeces	JCM
*B. breve* NCFB2257	Isolate from infant intestine	NCFB
*B. breve* NCFB2257-pBC1.2	NCFB2257 harbouring plasmid pBC1.2	This study
*B. breve* NCFB2257-pBC1.2-galA	NCFB2257 harbouring complementation construct pBC1.2-galA	This study
*B. breve* NCFB2258	Isolate from infant intestine	NCFB
*B. breve* LMG13208	Isolate from infant intestine	LMG
*B. breve* NCIMB11815	Isolate from infant intestine	NCTC
*B. breve* NCIMB8815	Isolate from human faeces	NCTC
*B. breve* NIZO658	Isolate from human faeces	NIZO
*B. breve* UCC2008	Isolate from infant intestine	UCC
*B. breve* UCC2009	Isolate from infant intestine	UCC
*B. breve* UCC2010	Isolate from human milk	UCC
*Lactococcus lactis* strains		
*L. lactis* NZ9000	MG1363, pepN::nisRK, nisin-inducible overexpression host	de Ruyter *et al*. (1996)
*L. lactis* NZ9000-pNZ-Bbr_0010	NZ9000 containing pNZ-lacZ2	This study
*L. lactis* NZ9000-pNZ-gosG	NZ9000 containing pNZ-gosG	This study
*L. lactis* NZ9000-pNZ-galG	NZ9000 containing pNZ-galG	O'Connell Motherway *et al*. (2011b)
*L. lactis* NZ9000-pNZ-lacZ	NZ9000 containing pNZ-lacZ	This study
		
Plasmids		
pAM5	pBC1-puC19-Tc^r^	Alvarez-Martín *et al*. (2007)
pBC1.2	pBC1-pSC101-Cm^r^	Alvarez-Martín *et al*. (2007)
pBC1.2-galA	pBC1-pSC101-Cm^r^ harbouring *gal*A including its own promoter	This study
pORI19	Em^r^, repA^−^, ori^+^, cloning vector	Law *et al*. (1995)
pORI19-tet-gosD	Internal 325 bp fragment of *bbr_0527* and tetW cloned in pORI19	This study
pORI19-tet-gosG	Internal 395 bp fragment of *bbr_0529* and tetW cloned in pORI19	This study
pORI19-tet-lacS	Internal 479 bp fragment of *bbr_1551* and tetW cloned in pORI19	This study
pORI19-tet-lacZ	Internal 609 bp fragment of *bbr_1552* and tetW cloned in pORI19	This study
pNZ8150	Cmr, nisin inducible translational fusion vector	Mierau and Kleerebezem (2005)
pNZ-lacZ2	Cmr, pNZ8150 derivative containing translational fusion of Bbr_0010 encoding DNA fragment to nisin inducible promoter	This study
pNZ-gosG	Cmr, pNZ8150 derivative containing translational fusion GosG encoding DNA fragment to nisin inducible promoter	This study
pNZ-galG	Cmr, pNZ8150 derivative containing translational fusion of GalG encoding DNA fragment to nisin inducible promoter	O'Connell Motherway *et al*. (2011b)
pNZ-lacZ	Cmr, pNZ8150 derivative containing translational fusion of LacZ encoding DNA fragment to nisin inducible promoter	This study

JCM, Japan Collection of Microorganisms; LMG, Belgian Co-ordinated Collection of Microorganisms; NCFB, National Collection of Food Bacteria; NCTC, National Collection of Type Cultures; UCC, University College Cork Culture Collection.

When growth was examined using PGOS as the sole carbohydrate source, *B. breve* UCC2003-galA, *B. breve* UCC2003-galC and *B. breve* UCC2003-galG reached lower final optical densities as compared with UCC2003 or indeed the other four insertion mutant strains (Fig. 4AIII). These data suggest key roles for the endogalactanase encoded by *gal*A, the ABC transport system specified by *gal*CDE and the β-galactosidase encoded by *gal*G in the metabolism of PGOS by *B. breve* UCC2003.

**Fig. 5:**
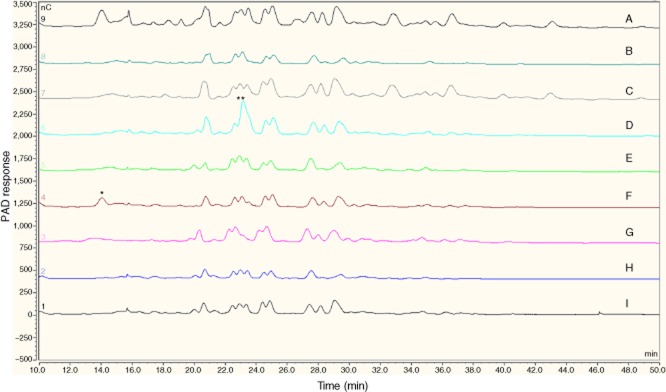
HPAEC-PAD analysis of post-fermentation cell-free supernatants of *B. breve* UCC2003 and insertion mutant strains. Modified Rogosa supplemented with 0.5% GOS (A); Post-fermentation supernatants of *B. breve* UCC2003 (B), UCC2003-galA (C), UCC2003-galC (D), UCC2003-galG (E), UCC2003-lacS (F), UCC2003-lacZ (G), UCC200-gosD (H), UCC2003-gosG (I). The position of lactose and galactotriose are indicated by asterisk (*) and double-asterisk (**) respectively.

**Fig. 6:**
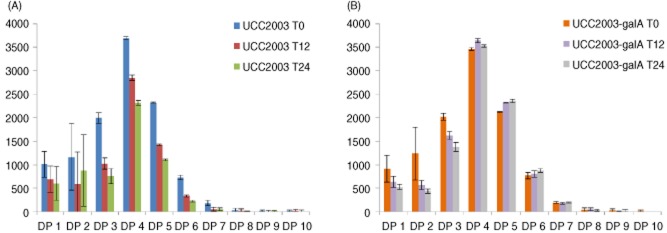
Mass Spectroscopy analysis of cell-free supernatants of *B. breve* UCC2003 (A) and *B. breve* UCC2003-galA (B). Samples were retained for analysis at 0, 12 and 24 h. Samples from duplicate experiments were run in triplicate and the data presented are averages of these six data sets with standard deviations.

HPAEC-PAD analysis of the cell-free supernatants identified that the *B. breve* UCC2003-galA strain did not metabolize PGOS components with a predicted high degree of polymerization (retention time 30–48 min) (Fig. 5C). This finding was verified by Mass Spectroscopy analysis that demonstrated that *B. breve* UCC2003 consumed all detectable PGOS fractions, while *B. breve* UCC2003-galA could only metabolize PGOS fractions with a DP of up to and equal to three (Fig. 6). Interestingly, a GOS component present in fractions eluted from 22.5 to 25 min, of the HPAEC analysis, was shown to increase in the CFS of *B. breve* UCC2003-galC (Fig. 5D). This GOS component is likely β,1-4 linked galactotriose, which is the main product of the endogalactanase activity (Hinz *et al*., 2005; O'Connell Motherway *et al*., 2011b), and which is apparently not transported by this strain due to the *gal*C mutation. When *B. breve* UCC2003-lacS was grown on PGOS, it was incapable of internalizing lactose, a GalA-mediated PGOS degradation product, which therefore accumulates in the growth medium, and which elutes at approximately 14 min under the conditions tested (Fig. 5F). The CFS post-fermentation profiles obtained following growth of UCC2003-galG (Fig. 5E), UCC2003-lacZ (Fig. 5G), or UCC2003-gosG (Fig. 5I) in PGOS-containing medium, closely resembled that of *B. breve* UCC2003, a result that was not unexpected given that GalG, LacZ and GosG are all intracellular β-galactosidases, while the extracellular endogalactanase activity and transport functions are fully functional in these mutant strains.

### Complementation of *B. breve* UCC2003-galA and *B. breve* NCFB2257

**Fig. 7:**
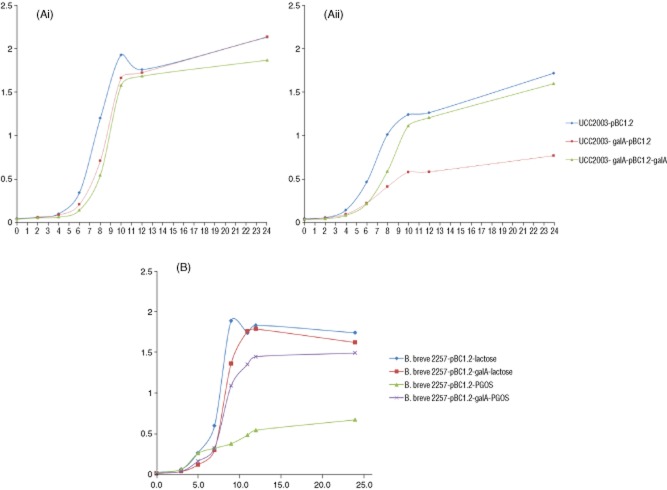
Growth profiles of (A) *B. breve* UCC2003 pBC1.2, UCC2003-galA-pBC1.2 and UCC2003-galA-pBC1.2-galA on lactose (Ai) and PGOS (Aii) and (B) *B. breve* NCFB2257-pBC1.2 and NCFB2257-pBC1.2-galA on lactose and PGOS. Data presented are averages of duplicate experiments.

To demonstrate that the protein product of *gal*A is uniquely necessary for the metabolism of galacto-oligosaccharides with a DP > 3, two complementation experiments were performed. In the first, the endogalactanase-encoding gene, *gal*A, was cloned under the control of its own promoter on plasmid pBC1.2 and introduced into *B. breve* UCC2003-galA (to generate strain UCC2003-galA-pBC1.2-galA; see *Experimental procedures*). Expression of GalA in strain UCC2003-galA-pBC1.2-galA allowed this strain to grow on PGOS to an optical density comparable to *B. breve* UCC2003 (Fig. 7Aii), while HPAEC analysis of the post-fermentation CFS of UCC2003-galA-pBC1.2-galA clearly shows the consumption of the PGOS fractions with a high degree of polymerization (Fig. S1). For the second complementation experiment plasmid pBC1.2-galA was introduced into the GalA^−^ strain *B. breve* NCFB2257 (to generate strain *B. breve* NCFB2257-pBC1.2-galA). Expression of GalA in *B. breve* NCFB2257-pBC1.2-galA enabled this strain to grow to a higher optical density compared with the wild type, *B. breve* NCFB2257, when grown on PGOS as the sole carbohydrate source. This PGOS-dependent growth profile of *B. breve* NCFB2257-pBC1.2-galA was comparable to those observed for GalA^+^ *B. breve* strains (Fig. 7B), while HPAEC analysis demonstrated that *B. breve* NCFB2257-pBC1.2-galA could metabolize PGOS components with a high degree of polymerization (Fig. S2). These complementation experiments unequivocally demonstrate the role of GalA in the metabolism of PGOS with a high degree of polymerization and long retention time.

## Discussion

Several studies have demonstrated that bifidobacteria dedicate a significant portion of their coding capacity to the metabolism of a wide variety of carbohydrates (Schell *et al*., 2002; Ventura *et al*., 2007a,b). Over 50 different bifidobacterial carbohydrases have been described in the literature to date (reviewed by Van den Broek *et al*., 2008; Pokusaeva *et al*., 2011a). Using *B. breve* UCC2003 as a model to study bifidobacterial carbohydrate metabolism, we previously characterized an operon encoding a β-fructofuranosidase (Ryan *et al*., 2005), an extracellular amylopullulanase which hydrolyses α-1,4 and α-1,6 glucosidic linkages in starch and related polysaccharides (Ryan *et al*., 2006; O'Connell Motherway *et al*., 2012), two α-glucosidases exhibiting hydrolytic activities towards panose, isomaltose, isomaltotriose and four sucrose isomers-palatinose, trehalulose, turanose and maltulose (Pokusaeva *et al*., 2009), as well as gene clusters dedicated to ribose (Pokusaeva *et al*., 2010), galactan (O'Connell Motherway *et al*., 2011b) and cellodextrin (Pokusaeva *et al*., 2011b) metabolism. In addition, a PEP-PTS system involved in fructose metabolism was identified and studied in this bacterium (Maze *et al*., 2007).

In this study, we describe the identification and functional characterization of genetic loci involved in the utilization of PGOS by *B. breve* UCC2003. To date several studies have demonstrated the presence of β-galactosidase isoenzymes in bifidobacteria (Van Laere *et al*., 2000; Barboza *et al*., 2009; Goulas *et al*., 2009; Osman *et al*., 2010); however, to our knowledge this is the first study adopting functional genomics approaches to understand the apparent synergistic relationship between β-galactosidases and their associated transport systems required for PGOS metabolism by bifidobacteria.

A range of *B. breve* strains was tested for their ability to grow on PGOS as the sole carbohydrate source, and while all strains were shown to grow well on PGOS, reaching final optical densities of ≥ 0.8, two distinct groups could be identified based on the growth profiles and analysis of the post-fermentation cell-free supernatants by HPAEC-PAD. The first group included *B. breve* UCC2003 and for this group HPAEC-PAD analysis showed that all strains metabolized most GOS components including those with a high degree of polymerization. Interestingly and consistent with our expectations, CGH analysis (O'Connell Motherway *et al*., 2011a) revealed that these strains each encoded an extracellular endogalactanase. The strains in the second group also grew well on PGOS but reached a lower final optical density; however, HPAEC-PAD analysis of the post-fermentation CFS clearly showed that oligosaccharides with a high degree of polymerization remained in the CFS of these strains and were not metabolized. This correlated with the CGH analysis where each of these strains was found to lack the *galA* gene. These data suggest a key role for GalA in the metabolism of galacto-oligosaccharides with a high degree of polymerization.

DNA microarrays that allowed comparison of gene expression of exponentially growing *B. breve* UCC2003 when grown on either PGOS or ribose as the sole carbohydrate source identified four gene clusters that are highly upregulated. These include the previously described galactan gene cluster, *gal*BCDEGRA (O'Connell Motherway *et al*., 2011b), as well as three additional clusters *gos*RDEGC, *lac*SZI and *gal*TK. Here we demonstrate that compared with the parent strain UCC2003, the *galA*, *galC* and *galG* mutant strains exhibited impaired growth on PGOS, thereby demonstrating that the endogalactanase, ABC transport system and β-galactosidase encoded by the *gal* locus significantly contribute to the extracellular metabolism, transport and subsequent intracellular metabolism of PGOS oligosaccharides respectively. Furthermore, analysis of the CFS of UCC2003-galA by HPAEC-PAD following growth on PGOS identified the presence of oligosaccharide fractions with a high degree of polymerization as observed for the galA^−^ *B. breve* strains and further supporting the importance of GalA in the metabolism of GOS oligosaccharide fractions with a high degree of polymerization. Our recent studies on starch (O'Connell Motherway *et al*., 2012) and cellodextrin (Pokusaeva *et al*., 2011b) metabolism by *B. breve* UCC2003 have demonstrated that the associated ABC transport systems can internalize malto-oligosaccharides or cellodextrins with a DP of 2–5. We would therefore expect that the *B. breve gal* and *gos* locus encoded ABC transport systems would transport PGOS fractions with similar relatively low degrees of polymerization and that utilization of GOS oligosaccharide fractions with a high degree of polymerization requires initial extracellular degradation by endogalactanase. This expectation was confirmed by Mass Spectroscopy analysis which demonstrated that GalA enables bifidobacterial cells to metabolize PGOS components with a DP ≥ 4. It is plausible that other *Bifidobacterium* sp. may possess an extracellular β-galactosidase that may fulfil a similar role; however, to date just two extracellular β-galactosidases from bifidobacteria have been described in the literature: while Moller and colleagues (2001) did not evaluate the ability of the extracellular β-galactosidase, BIF3, from *B. bifidum* DSM20215 to hydrolyse GOS, BbgIII from *B. bifidum* NCIMB41171 was found to have a high affinity for lactose hydrolysis but exhibited relatively low GOS hydrolysis activity (Goulas *et al*., 2009).

Our results also demonstrate a significant role for the LacS symporter in transporting lactose, lactulose and possibly other GOS di-/oligo-saccharides into *B. breve* UCC2003. Collectively these data demonstrate that PGOS metabolism by *B. breve* UCC2003 can be attributed to the cooperative activity of multiple β-galactosidases and their associated transport systems. Our observations are consistent with those of Barboza and colleagues (2009) who examined PGOS consumption of four bifidobacterial strains, namely *B. breve* ATCC15700, *B. longum* subsp. *infantis* ATCC15697, *B. adolescentis* DSM20083 and *B. longum* subsp. *longum* DJ010A, and observed strain specific preferences for PGOS utilization. Interestingly, and in contrast to our findings for *B. breve* UUCC2003, Andersen and colleagues (2011) have recently reported that LacS is the sole transporter of lactose, lactulose, lactitol and GOS into *Lactobacillus acidophilus* NCFM although it has yet to be demonstrated if *L. acidophilus* NCFM can utilize GOS components with a high degree of polymerization.

The ability of probiotic strains to ferment particular oligo- and poly-saccharides has been the basis for their selection as prebiotics and incorporation into functional foods. The health benefits associated with GOS are well documented and many placebo controlled clinical trials have explored the health benefits of incorporating GOS in functional food formulations. It is now well documented that GOS promotes the proliferation of beneficial bacteria, in particular bifidobacteria, in the gut, which can subsequently provide protection against colonization with pathogens thereby reducing the incidence of infections. In addition, metabolism of GOS by the gut microbiota leads to the production of short-chain fatty acids (SCFAs). These SCFAs are reported to have numerous beneficial health effects that include reducing cancer risk, increased mineral absorption, improving bowel habit, stool consistency and reduction of IBD inflammation (Davis *et al*., 2011 reviewed by MacFarlane *et al*., 2008; Sangwan *et al*., 2011).

Future research will focus on characterizing the specific PGOS components that are metabolized by *B. breve* UCC2003, in particular identifying the PGOS structures that are metabolized by GalA, identifying the GOS components that are transported by the ABC-transport systems and LacS. It will also be necessary to determine if differences exist in substrate specificities of the identified β-galactosidases. The results presented here significantly advance our knowledge of GOS metabolism by bifidobacteria and, in due course, may contribute towards the development of targeted bifidogenic galacto-oligosaccharides, for specific probiotic strains. The incorporation of such galacto-oligosaccharides in foods has potential for the development of enhanced functional foods or infant food formulas.

## Experimental procedures

The description of the *Experimental procedures* resides in Appendix S1 in *Supporting information*.

## Supporting information

**Fig. S1.** HPAEC-PAD analysis of post fermentation cell free supernatants of *B. breve* UCC2003, UCC2003-gal A and complemented strain. Modified Rogosa supplemented with 0.5 % GOS (A); post-fermentation supernatants of *B. breve* UCC2003 (B), UCC2003-galA-pBC1.2 (C) and UCC2003-galA-pBC1.2-galA (D).

**Fig. S2.** HPAEC-PAD analysis of post fermentation cell free supernatants of *B. breve* NCFB2257-pBC1.2, and GalA complemented derivative. Modified Rogosa supplemented with 0.5 % GOS (A); post-fermentation supernatants of *B. breve* NCFB2257-pBC1.2 (B) and *B. breve* NCFB2257-pBC1.2-galA (C).

**Appendix S1.** Experimental procedures.
