# Supplementary material for: Transcriptional and functional characterization of genetic elements involved in galacto-oligosaccharide utilization by Bifidobacterium breve UCC2003
Source: Microb Biotechnol. 2012 Dec 2;6(1):67–79. doi: 10.1111/1751-7915.12011 (PMC3815386; doi:10.1111/1751-7915.12011)
Supplement: Supplementary file 1 [file mbt0006-0067-SD1.ppt]

## Slide 1
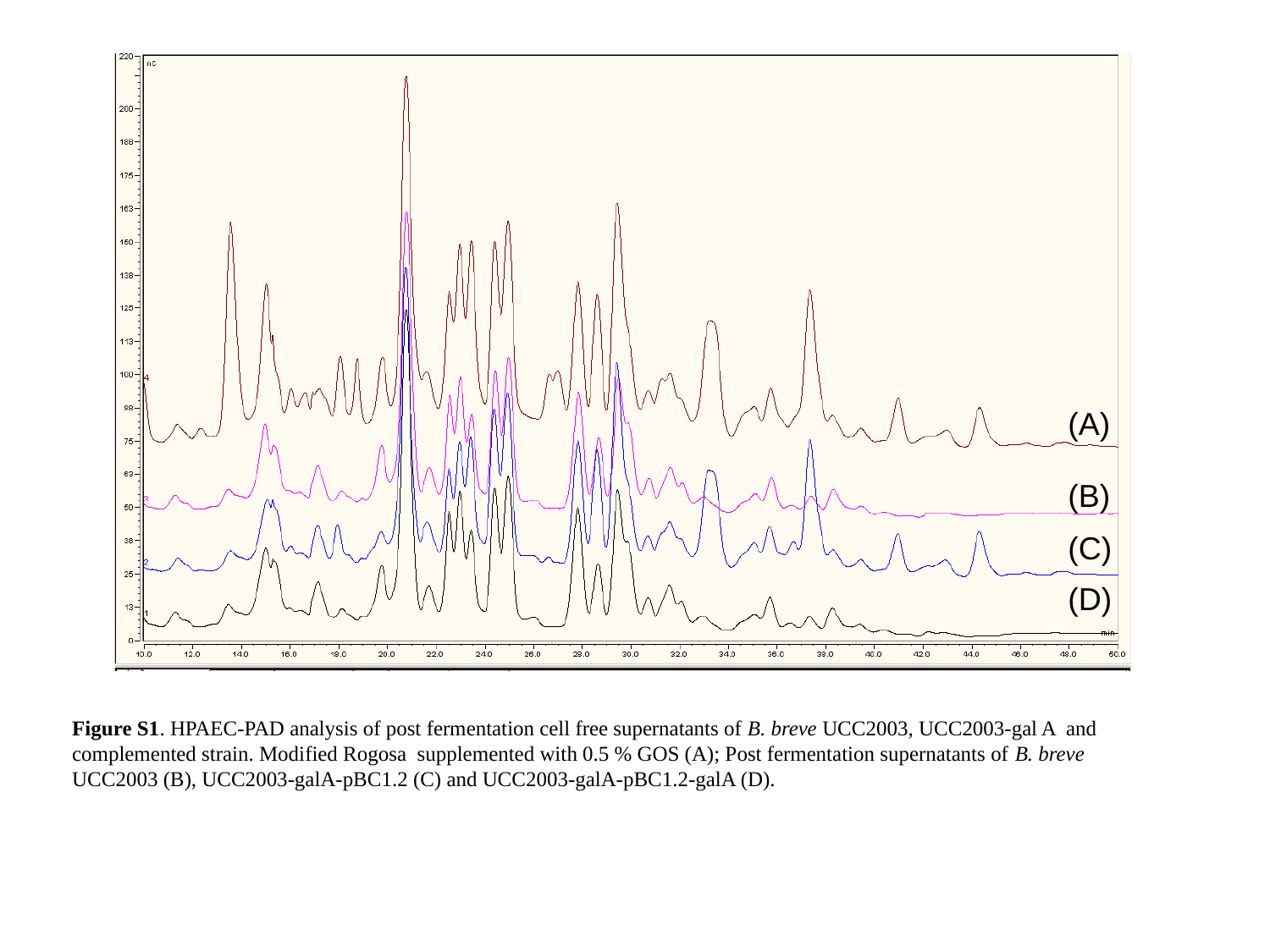

(A)
(B)
(C)
(D)
Figure S1. HPAEC-PAD analysis of post fermentation cell free supernatants of B. breve UCC2003, UCC2003-gal A and complemented strain. Modified Rogosa supplemented with 0.5 % GOS (A); Post fermentation supernatants of B. breve UCC2003 (B), UCC2003-galA-pBC1.2 (C) and UCC2003-galA-pBC1.2-galA (D).

## Slide 2
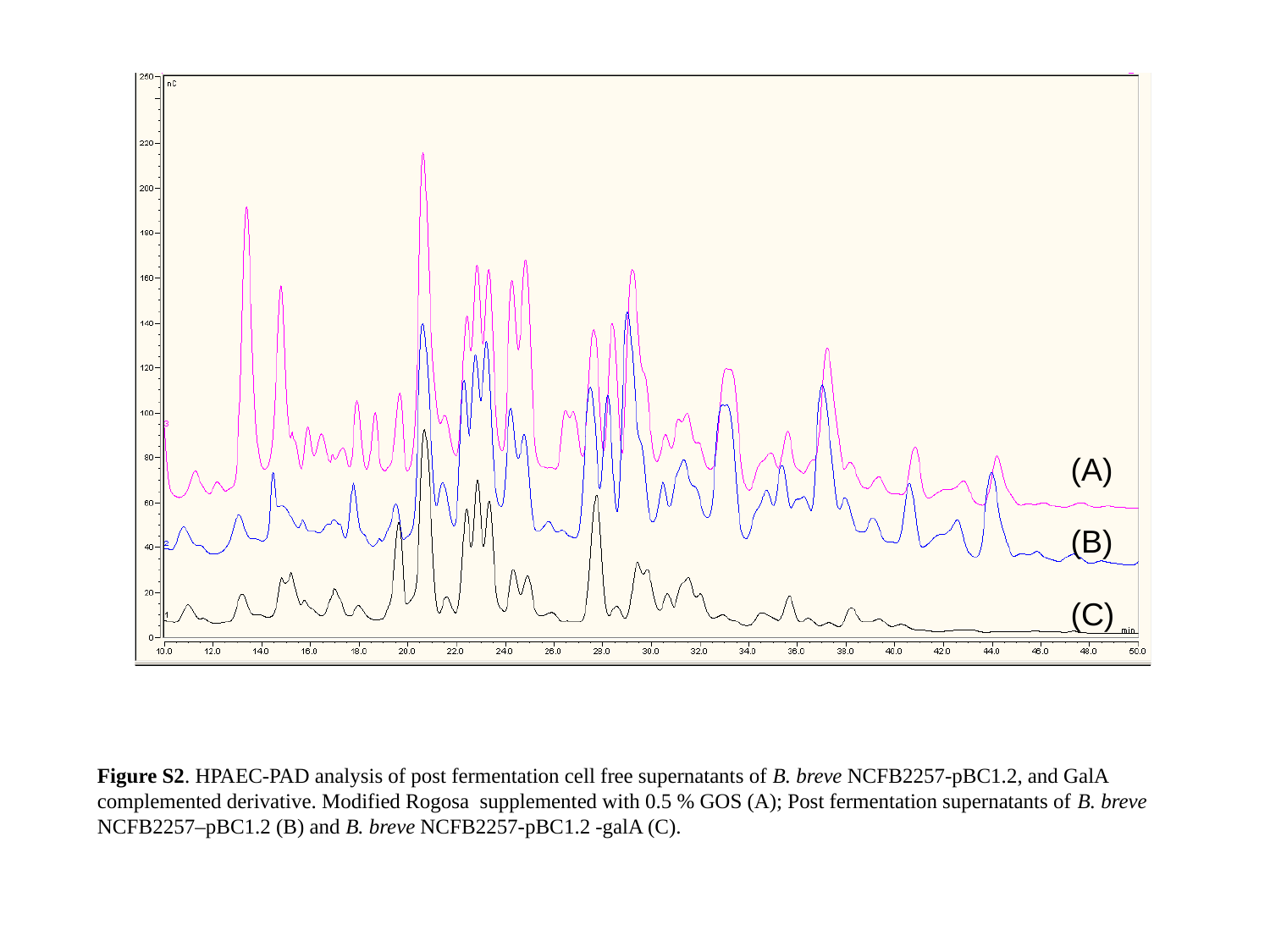

(A)
(B)
(C)
Figure S2. HPAEC-PAD analysis of post fermentation cell free supernatants of B. breve NCFB2257-pBC1.2, and GalA complemented derivative. Modified Rogosa supplemented with 0.5 % GOS (A); Post fermentation supernatants of B. breve NCFB2257–pBC1.2 (B) and B. breve NCFB2257-pBC1.2 -galA (C).
