# Supplementary material for: Transcriptional and functional characterization of genetic elements involved in galacto-oligosaccharide utilization by Bifidobacterium breve UCC2003
Source: Microb Biotechnol. 2012 Dec 2;6(1):67–79. doi: 10.1111/1751-7915.12011 (PMC3815386; doi:10.1111/1751-7915.12011)
Supplement: Supplementary file 2 [file mbt0006-0067-SD2.doc]

**Appendix S1**

**Experimental procedures**

**Bacterial strains, plasmids and culture conditions.** Bacterial strains and plasmids used in this study are listed in Table 1. Bifidobacterial strains were routinely cultured in reinforced clostridial medium (RCM; Oxoid Ltd, Basingstoke, Hampshire, United Kingdom). Carbohydrate utilization by bifidobacteria was examined in de Man Rogosa and Sharpe Medium (mMRS) prepared from first principles (de Mann, Rogosa and Sharpe, 1960). Prior to inoculation MRS was supplemented with cysteine-HCl (0.05 % w/v) and a particular carbohydrate source (0.5% w/v). The carbohydrates used were PGOS (obtained from Vivinal® GOS, FrieslandCampina DOMO, Amersfoort, The Netherlands, and containing 98.5 % GOS, 0.7 % galactose, 1.6 % glucose and 0.7 % lactose), lactose, lactulose and ribose (all purchased from Sigma). Bifidobacterial cultures were incubated at 37 ^o^C under anaerobic conditions which were maintained using an anaerobic hood (Davidson and Hardy, Belfast, Ireland). *Escherichia coli* was cultured in Luria Bertani broth (LB) (Sambrook *et al*., 1989) at 37 ^o^C with agitation while *L. lactis* was grown in M17 broth (Oxoid, UK) supplemented with 0.5 % glucose at 30 ^o^C. Where appropriate growth media contained ampicillin (Amp; 100 μg ml^-1^), tetracycline (Tet; 15 μg ml^-1^), chloramphenicol (Cm; 5 μg ml^-1^ for *E.coli and L. lactis*), erythromycin (Em; 100 μg ml^-1^ for *E. coli*) or kanamycin (Km; 50 μg ml^-1^ for *E. coli*). Recombinant *E. coli* cells containing pORI19 or pBC1.2 (or derivatives thereof) were selected on LB agar containing Em or Amp, respectively, and supplemented with X-gal (5-bromo-4-chloro-3-indolyl-β-D-galactopyranoside) (40 μg ml^-1^) and 1 mM IPTG (isopropyl-β-D-galactopyranoside).

**Nucleotide sequence analysis.** Sequence data were obtained from the Artemis-mediated (Rutherford *et al.,* 2000) genome annotations of the *B. breve* UCC2003 (O’Connell Motherway *et al.,* 2011). Database searches were performed using non-redundant sequences accessible at the National Centre for Biotechnology Information internet site ([http://www.ncbi.nlm.nih.gov](http://www.ncbi.nlm.nih.gov/)) using Blast.

**DNA manipulations**. Chromosomal DNA was isolated from *B. breve* UCC2003 as previously described (O’Riordan, 1998). Minipreparation of plasmid DNA from *E. coli* or *L. lactis* was achieved using the Qiaprep spin plasmid miniprep kit (Qiagen GmBH, Hilden, Germany). For *L. lactis* an initial lysis step was included whereby cells are incubated in lysis buffer containing 30 mg ml^-1^ of lysozyme for 30 minutes at 37 ^o^C. Single stranded oligonucleotide primers used in this study were synthesized by Eurofins (Ebersberg, Germany). Standard PCRs were performed using TaqPCR mastermix (Qiagen), while high fidelity PCR was achieved using KOD polymerase (Novagen, Darmstadt, Germany). *B. breve* colony PCRs were performed as described previously (O’Connell Motherway *et al*., 2008). PCR fragments were purified using the Qiagen PCR purification kit (Qiagen). Electroporation of plasmid DNA into *E. coli* was performed as described by Sambrook *et al*. (1989), into *L. lactis* as described by Wells *et al*. (2003) and into *B. breve* strains as described by Maze *et al*. (2007).

**Analysis of global gene expression using *B. breve* DNA microarrays.** DNA-microarrays containing oligonucleotide primers representing each of the 1864 annotated genes on the genome of *B. breve* UCC2003 were designed by and obtained from Agilent Technologies (Palo Alto, Ca., USA). Methods for cell disruption, RNA isolation, RNA quality control, complementary DNA synthesis and labelling were performed as described previously (Pokusae*va et* al., 2009). Labelled cDNA was hybridized using the Agilent Gene Expression hybridization kit (part number 5188-5242) as described in the Agilent Two-Color Microarray-Based Gene Expression Analysis v4.0 manual (G4140-90050). Following hybridization, microarrays were washed in accordance with Agilent’s standard procedures and scanned using an Agilent DNA microarray scanner (model G2565A). Generated scans were converted to data files with Agilent's Feature Extraction software (Version 9.5). DNA-microarray data were processed as previously described (Garcia De La Na*va et* al., 2003). Differential expression tests were performed with the Cyber-T implementation of a variant of the *t*-test (Lo*ng et* al., 2001). A gene was considered differentially expressed when p < 0.001 and an expression ratio of >3 or <0.33 relative to the control. The microarray data have been deposited in NCBI's Gene Expression Omnibus and are accessible through GEO Series accession number GSE37214.

**Construction of *B. breve* UCC2003 insertion mutants.** Internal fragments of the open reading frames Bbr_0527 (designated here as *gos*D), Bbr_0529 (designated *gos*G), Bbr_1551 (designated *lac*S*)* and bbr_1552 (designated *lac*Z*)* (corresponding to codon numbers 78 through to 153 out of the 308 codons present in Bbr_0527; codons 179 through to 309 of the 691 codons of Bbr_0529; codons 133 through to 293 of the 493 codons present in Bbr_1551*;* codons 263 through to 466 of the 1045 codons present in Bbr_1552) were amplified by PCR using *B. breve* UCC2003 chromosomal DNA as a template and primer pairs IM527F and IM527R, IM529F and IM529R, IM1551F and IM1551R or IM1552F and IM1552R, respectively (Table S1). Insertion mutations in *gos*D, *gos*G, *lac*S and *lac*Z genes were generated essentially as described previously (O’Connell Motherway *et al*., 2009) to produce *B. breve* UCC2003 derivatives that were designated UCC2003-gosD, UCC2003-gosG*,* UCC2003-lacS and UCC2003-lacZ, respectively. Site-specific recombination in potential tet-resistant mutant isolates was confirmed by colony PCR using primer combinations tetWFw and tetWRv to verify *tetW* gene integration, and primers 527-FW, 529-FW, 1551-FW or 1552-FW (positioned upstream of the selected internal fragment of *gos*D, *gos*G, *lac*S or *lac*Z, respectively), each in combination with pORI19For to confirm integration at the correct chromosomal location.

**Analysis of carbohydrate utilization by High performance Anion exchange chromatography with Pulse Amperometric Detection.** The oligosaccharide profiles of post-fermentation cell free supernatants, collected at 24h, were determined using High-Performance Anion Exchange Chromatography with Pulsed Amperometric Detection (HPAEC-PAD). For this purpose a Dionex ICS-3000 system (Dionex, Sunnyvale, CA) was used. Oligosaccharide fractions (25 μl aliquots) were separated on a CarboPac PA1 (Dionex) analytical-exchange column with dimensions 250 mm x 4 mm with a CarboPac PA1 guard column (Dionex) with dimensions 50 mm x 4 mm and a pulsed electrochemical detector in the pulsed amperometric detection (PAD) mode. The elution was performed at a constant flow-rate of 1.0 ml min^-1^ at 30°C using the following eluents for the analysis (A) 200 mM NaOH, (B) 100 mM NaOH, 550 mM NaAC, and (C) Milli-Q water. The following linear gradient of sodium acetate was used: 100 mM NaOH: 0-50 min, 0 mM; 50-51 min, 16 mM; 51-56 min, 100 mM; 56.1-61 min, 0 mM. Detection was achieved using a Dionex ED40 detector in pulsed amperometric detection mode. Chromatographic profile of non-fermented GOS was used for comparison of the results on GOS utilization from samples and the Chromeleon software-Version 6.70 (Dionex Corporation) was used for the integration and evaluation of the chromatograms obtained.

**Liquid Chromatography Mass Spectroscopy analysis.** Mass spectrometry analysis was conducted using a Waters Acquity G2 Q-TOF LC-MS instrument. This system is composed of a Waters Acquity UPLC system coupled to a Quadrupole Time-of flight mass spectrometer. The GOS samples were eluted using a Waters Acquity UPLC BEH C18 column, 1.7 µm particle size, 2.1 x 100 mm with the column temperature held at 40 °C and samples refrigerated to 4 °C in the UPLC autosampler. All solvents used were LC-MS grade and ultra pure 18.2 MΩ water was used for each step. A gradient of water:acetonitrile A:B and a 10 µL injection volume was used to elute the sample. Initially, 98:2 A:B was held until 2.5 minutes followed by a linear increase to 98% B after 4 minutes. After a further 2 minutes, the system was returned back to initial conditions (98:2 A:B) giving a total run time of 7 minutes. The mass spectrometry detection was conducted by electrospray ionisation in negative mode in the m/z scan range 100-3000 Da using the following settings: Capillary voltage 2.5 kV, Sampling cone 30 V, Extraction Cone 3 V, Source temperature 120 °C, Desolvation temperature 500 °C, Desolvation gas flow 1200 L/hour, Cone gas flow 10 L/hour. The accurate mass of the instrument was initially calibrated through direct infusion of a sodium iodide calibrant solution and during the experiment, leucine enkephalin (Leuenk) lockmass solution (2 ng/uL) was infused at 5 µL/min in parallel to the mobile phase flow. The exact mass of the Leuenk parent M-H ion served to continuously monitor and correct the exact mass detected and calculated by the instrument. The peak areas of each saccharide molecule were calculated using Waters Targetlnx quantitation software.

**Plasmid Constructions**. For the construction of plasmids pNZ-lacZ2, pNZ-gosG and pNZ-lacZ, DNA fragments encompassing the predicted β-galactosidase-encoding genes lacZ2 (Bbr_0010)*, gosG* (Bbr_0529) or lacZ (Bbr_1552) were generated by PCR amplification from chromosomal DNA of *B. breve* UCC2003 using KOD DNA polymerase and primer combinations Bbr_0010F and Bbr_0010R, gosGF and gosGR, and lacZF and lacZR, respectively. EcoRV and XbaI restriction sites were incorporated at the 5’ ends of each forward and reverse primer combination, respectively (Table S1). In addition, an in frame His_10_-encoding sequence was incorporated into each of the forward primers to facilitate downstream protein purification using the Ni-NTA affinity system (Qiagen). The three generated amplicons were digested with EcoRV and XbaI, and ligated into ScaI and XbaI-digested nisin-inducible translational fusion plasmid pNZ8150 (Mierau and Kleerebezem, 2005). The ligation mixtures were introduced into *L. lactis* NZ9000 (Table 1) by electrotransformation and transformants selected based on chloramphenicol resistance. For the construction of the complementation construct pBC1.2-galA, a DNA fragment encompassing *gal*A, including its native promoter region was generated by PCR amplification from chromosomal DNA of *B. breve* UCC2003 using PFUII DNA polymerase (Agilent) and primer combination galAF and galAR (see Table S1). The *galA*-containing amplicon was digested with EcoRI and XbaI, and ligated to similarly digested PBC1.2. The ligation was introduced into *E. coli*XL1-Blue by electroporation. For all cloning experiments, the plasmid content of a number of transformants was screened by restriction analysis and the integrity of positively identified clones was verified by sequencing.

**Protein overproduction and β-galactosidase assays**. 30 ml of M17 broth supplemented with 0.5 % glucose was inoculated with a 2 % inoculum of a particular *L. lactis* strain, followed by incubation at 30 ^o^C until an Optical Density (O.D. at wavelength 600 nm) of 0.5 was reached, at which point each culture was divided in two and protein expression was induced by the addition of purified nisin (5 ng ml^-1^) to one volume of each culture and incubation was continued at 30 ^o^C for 90 minutes. Cells were harvested by centrifugation and β-galactosidase activity was determined as described previously (Israelsen *et al*., 1995).

**References**

De Man, J. C., Rogosa A. and Sharpe, M. E. (1960) A medium for the cultivation of lactobacilli. *J Appl Bacteriol* **23**: 130-135.

Garcia De La Nava, J., D., Santaella, F., Alba, J. C., Carazo, J. M., Trelles O., and Pascual-Montano, A. (2003) Engene: the processing and exploratory analysis of gene expression data. *Bioinformatics* **19**: 657-658.

Israelsen, H., Madsen, S.M., Vrang, A., Hansen, E.B., and Johansen, E. (1995) Cloning and partial characterization of regulated promoters from *Lactococcus lactis* Tn917-lacZ integrants with the new promoter probe vector, pAK80. *Appl Environ Microbiol* **61**(7): 2540-7.

Long, A. D., Mangalam, H. J., Chan, B. Y., Tolleri, L., Hatfield G. W., and Baldi, P. (2001) Improved statistical inference from DNA microarray data using analysis of variance and a Bayesian statistical framework. Analysis of global gene expression in *Escherichia coli*  K12. *J Biol Chem* **276**: 19937-19944.

Maze, A., O'Connell-Motherway, M., Fitzgerald, G. F., Deutscher J., and Van Sinderen, D. (2007) Identification and characterization of a fructose phosphotransferase system in *Bifidobacterium breve*  UCC2003. *Applied and Environmental Microbiology* **73**: 545-553.

Mierau, I., and Kleerebezem, M. (2005) 10 years of the nisin-controlled gene expression system (NICE) in Lactococcus lactis. *Appl Microbiol Biotechnol*. **68**: 705-17.

O'Connell Motherway, M., Zomer, A., Leahy, S.C., Reunanen, J., Bottacini, F., Claesson, M.J., et al. (2011) Functional genome analysis of *Bifidobacterium breve* UCC2003 reveals type IVb tight adherence (Tad) pili as an essential and conserved host-colonization factor. *Proc Natl Acad Sci U S A.* **108** (27):11217-22.

O'Connell Motherway, M., O' Driscoll, J., Fitzgerald, G.F., and Van Sinderen, D. (2009) Overcoming the restriction barrier to plasmid transformation and targeted mutagenesis in *Bifidobacterium breve* UCC2003. *Microb Biotechnol* **2**(3): 321-32.

O'Riordan, K. (1998) Studies on antimicrobial activity and genetic diversity of Bifidobacterium species: molecular characterization of a 5.75 kb plasmid and a chromosomally encoded *rec*A gene homologue from *Bifidobacterium breve*. Ph.D. National University of Ireland, Cork, Cork.

Pokusaeva, K., O'Connell-Motherway, M., Zomer, A., Fitzgerald G. F., and Van Sinderen, D. (2009) Characterization of two novel alpha-glucosidases from *Bifidobacterium breve* UCC2003. *Appl Environ Microbiol* **75**: 1135-1143.

Rutherford, K., Parkhill, J., Crook, J., Horsnell, T., Rice, P., Rajandream, M.A., and Barrell, B. (2000). Artemis: sequence visualization and annotation. *Bioinformatics* **10**: 944-945.

Sambrook, J., Fritsch, E. F., and Maniatis, T. (1989). Molecular cloning a laboratory manual, 2nd ed. Cold Spring Harbor Laboratory, Cold Spring Harbor, N.Y.

Wells, J.M., Wilson, P.W. and Le Page, R.W. (1993) Improved cloning vectors and

transformation procedure for *Lactococcus lactis*. *J Appl Bacteriol* **74**: 629-36.

**Table S1.Oligonucleotide Primers used in this Study**

| ***Purpose*** | ***Primer*** | ***Sequence^a^*** |
| --- | --- | --- |
| Cloning of internal 325bp fragment of *gos*D in pORI19 | IM527F | tgcgg**aaagctt**cagttctggtcgtccgtg |
|  | IM527R | ctatgc**tctaga**gcatgttgtagccggtga |
|  |  |  |
| Cloning of internal 395bp fragment of *gos*G in pORI19 | IM529F | tgcgga**aagctt**cagaagtggtgcgagaagaag |
|  | IM529R | ctatgc**tctaga**gcgatgccgtcacacag |
|  |  |  |
| Cloning of internal 479bp fragment of *lac*S in pORI19 | IM1551F | tgcgga**aagctt**gcgtgttcatggtcctgttcgtc |
|  | IM1551R | ctatgc**tctaga**gaactggtagaagagcacgccg |
|  |  |  |
| Cloning of internal 609bp fragment of *lac*Z in pORI19 | IM1552F | ctggtc**aagctt**tcgctctcgctggacatac |
|  | IM1552R | cgagct**tctaga**gatcatgctgtcgagtcg |
|  |  |  |
| Complementation of *B. breve* UCC2003-galA and *B. breve* NCFB 2257 | galAF | tagca**gaattc**cgaactgctcaccaccgaatc |
|  | galAR | gata**tctctaga**catccaagcagacgaaaaccgac |
| Amplification of TetW | tetWF  tetWR | Atgctcatgtacggtaag  cattaccttctgaaacata |
|  |  |  |
|  | pORI19 for | attgtgagcggataacaatttcac |
| Confirmation of site specific homologous recombination | 1551-FW | gatccaccacacagcgcatg |
|  | 1552-FW | atgaacacaaccgacgatc |
|  | 527-FW | atgtcgctgccgtgcctatg |
|  | 529-FW | caatggcctgaggaagtgtg |
|  |  |  |
| Cloning of Bbr_0010 in pNZ8150 | Bbr_0010F | tgcatc**gatatc**atgcatcaccatcaccatcaccatcaccatcacatgaacttgcagtcagctc |
|  | Bbr_0010R | tgcgca**tctaga**tcagatgagttcgagtgtcac |
|  |  |  |
| Cloning of *gos*G in pNZ8150 | gosGF | tgcatc**gatatc**atgcatcaccatcaccatcaccat caccatcacatggaacatcgcgaattcaag |
|  | gosGR | tgcgca**tctaga**ttacagctttaccaccagcac |
|  |  |  |
|  |  |  |
| Cloning of *lac*Z in pNZ8150 | lacZF | tgcatc**gatatc**atgcatcaccatcaccatcaccat caccatcacatgaacacaaccgacgatcag |
|  | lacZR | tgcgca**tctaga**tcagatgagttcgaggttcac |

^a^ Restriction sites incorporated into oligonucleotide primer sequences are indicated in bold
